# Pharmacokinetic Effects of *l*-Tetrahydropalmatine on Ketamine in Rat Plasma by Ultraperformance Liquid Chromatography Tandem Mass Spectrometry

**DOI:** 10.1155/2020/9259683

**Published:** 2020-07-06

**Authors:** Yan Du, Hongliang Su, Jie Cao, Zhiwen Wei, Yujin Wang, Keming Yun

**Affiliations:** ^1^School of Pharmaceutical Science, Shanxi Medical University, Taiyuan, 030001 Shanxi, China; ^2^School of Pharmacy, University of Maryland Baltimore, Baltimore, 21201 MD, USA; ^3^School of Forensic Medicine, Shanxi Medical University, Taiyuan, 030001 Shanxi, China

## Abstract

Male Sprague-Dawley rats (*n* = 18) were randomly divided into three groups: a saline group (20 mL/kg by gavage), a ketamine (KET) group (100 mg/kg by gavage), and a KET (the same routes and doses) combined with *levo*-tetrahydropalmatine (*l*-THP; 40 mg/kg by gavage) group (*n* = 6). Blood samples were acquired at different time points after drug administration. A simple and sensitive ultraperformance liquid chromatography tandem mass spectrometry (UPLC-MS/MS) method was established to determine the concentrations of KET and its metabolite, norketamine (NK), in rat plasma. Chromatographic separation was achieved using a BEH C18 column (2.1 mm × 50 mm, 1.7 *μ*m) with chlorpheniramine maleate (Chlor-Trimeton) as an internal standard (IS). The initial mobile phase consisted of acetonitrile–water with 0.1% methanoic acid (80 : 20, *v*/*v*). The multiple reaction monitoring (MRM) modes of m/z 238.1→m/z 179.1 for KET, m/z 224.1→m/z 207.1 for NK, and m/z 275→m/z 230 for Chlor-Trimeton (IS) were utilized to conduct a quantitative analysis. Calibration curves of KET and NK in rat plasma demonstrated good linearity in the range of 2.5–500 ng/mL (*r* > 0.9994), and the lower limit of quantification (LLOQ) was 2.5 ng/mL for both. Moreover, the intra- and interday precision relative standard deviation (RSD) of KET and NK were less than 4.31% and 6.53%, respectively. The accuracies (relative error) of KET and NK were below -1.41% and -6.07%, respectively. The extraction recoveries of KET and NK were more than 81.23 ± 3.45% and 80.42 ± 4.57%, respectively. This sensitive, rapid, and selective UPLC-MS/MS method was successfully applied to study the pharmacokinetic effects of *l-*THP on KET after gastric gavage. The results demonstrated that *l-*THP could increase the bioavailability of KET and promote the metabolism of KET. The results showed that *l-*THP has pharmacokinetics effects on KET in rat plasma.

## 1. Introduction

Ketamine (KET), originally used as a surgical anesthetic, became a popular street drug in the USA in the 1970s. KET abuse has spread over North America and Europe and has also affected China in recent years [1]. Based on its dissociative anesthetic properties, KET generates visual and auditory distortions and a sense of floating and dissociation in abusers [2]. The main metabolic pathway involves *N*-demethylation of KET by cytochrome (CYP) 4503A to form the active metabolite, norketamine (NK) [3].

*Levo*-tetrahydropalmatine (*l-*THP), an alkaloid isolated from the traditional Chinese herbal medicine *Corydalis* and *Stephania*, has been widely used in many traditional Chinese herbal preparations for around 40 years [4]. As a nonopioid analgesic, *l-*THP has analgesic, sedative, and anxiolytic properties. Pharmacological studies demonstrated that *l-*THP is an antagonist of dopamine (DA) D1 and D2 receptors [5]. The DA system has been reported to play an important role in drug addiction. As a nonselective DA antagonist, *l-*THP has recently emerged as a promising agent for treating addiction to many types of drugs, including cocaine [6, 7], methamphetamine [8], and oxycodone [9]. In our previous study, *l-*THP showed a potential therapeutic effect on KET addiction [10]. Moreover, *l-*THP is safe and effective, with no obvious side effects. However, the pharmacokinetic effects of *l-*THP on KET remain unclear.

In this study, a sensitive ultraperformance liquid chromatography tandem mass spectrometry (UPLC-MS/MS) method was developed to determine KET and NK concentrations in rat plasma. The pharmacokinetic effects of *l-*THP on KET were investigated to explore the anti-KET addiction mechanism of *l-*THP.

## 2. Materials and Method

### 2.1. Chemicals and Reagents

**Figure 1:**
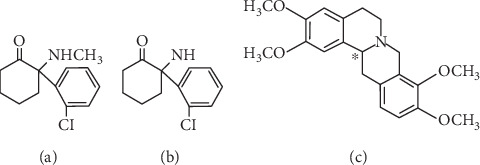
Molecular structure of ketamine (KET) (a), norketamine (NK) (b), and chlorpheniramine maleate (Chlor-Trimeton, IS) (c).

All reagents and solvents were of analytical grade. KET (purity > 98%, Figure 1(a)), NK (purity > 98%, Figure 1(b)), and chlorpheniramine maleate (Chlor-Trimeton; IS, purity > 98%, Figure 1(c)) standards were purchased from Chinese Pharmaceutical and Biological Products Institute (Beijing, China). KET hydrochloride was purchased from Hengrui Pharmaceutical Factory (Jiangxi, China). *l-*THP (99.00%) and UPLC-grade acetonitrile were purchased from Sigma-Aldrich Inc. (St. Louis, MO, USA). Male Sprague-Dawley rats (220–250 g) were purchased from Academy of Military Medical Science (AMMS, Beijing, China).

### 2.2. Instrumentation and Conditions

A UPLC-MS/MS system with TSQ Quantum (Waters, Xevo, TQS, USA) and Access Masslynx 4.1 software (Waters Corp.) was used to acquire the data and control instrument. Chromatographic separation was performed on a UPLC BEH C18 column (2.1 mm × 50 mm, 1.7 *μ*m) (Waters, Milford, MA USA) thermostatted at 25°C with a mobile phase composed of acetonitrile–water with 0.1% methanoic acid (80 : 20, *v*/*v*) at a flow rate of 0.2 mL/min. Before use, the mobile phase was filtered through a 0.45 *μ*m nylon membrane filter. The injection volume was 10 *μ*L, and the analysis time was 5 minutes for each sample.

**Figure 2:**
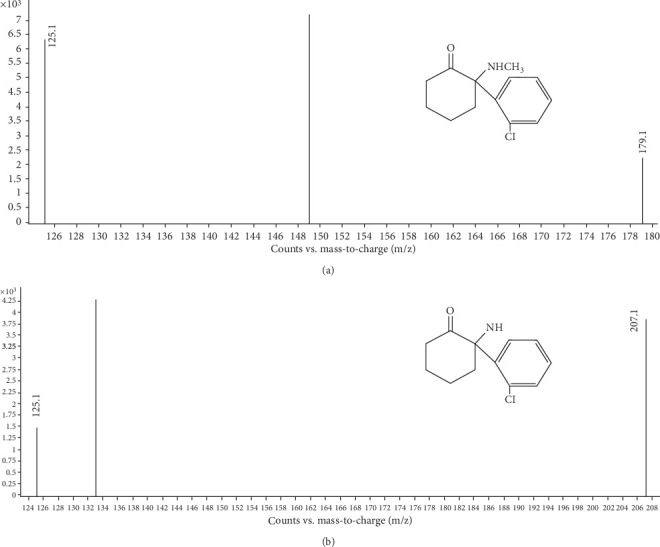
Second level mass spectrum of KET (a) and NK (b).

Nitrogen was used as a drying gas for solvent evaporation (40 L/h). The ion monitoring conditions were as follows: source temperature, 300°C and desolvation temperature, 110°C. The collision energy was 25 eV for KET, 19 eV for NK, and 25 eV for Chlor-Trimeton (IS). Quantification was performed using multiple reaction monitoring (MRM), with modes of m/z 238.1→m/z 179.1 for KET, m/z 224.1→m/z 207.1 for NK, and m/z 275→m/z 230 for Chlor-Trimeton (IS), Figure 2. The MRM parameters for KET, NK, and Chlor-Trimeton (IS) are presented in Table 1.

### 2.3. Preparation of Stock Solution, Calibration Standards, and Quality Control of Samples

KET, NK, and Chlor-Trimeton (IS) stock solutions were prepared in acetonitrile at a concentration of 1.0 mg/mL. Working solutions for calibration and controls were obtained by diluting the stock solutions with acetonitrile. All solutions were stored at 4°C until further use. Calibration standard samples were prepared by adding the KET and NK standards in various concentrations to blank rat plasma. Calibration plots were created for KET and NK in rat plasma (amounts of 2.5, 10, 12.5, 50, 100, 250, and 500 ng/mL). Quality control (QC) samples were prepared in the same manner as the calibration standards which is of low-quality control (LQC, 5 ng/mL), medium-quality control (MQC, 50 ng/mL), and high-quality control (HQC, 500 ng/mL), respectively.

### 2.4. Plasma Sample Preparation

Plasma sample aliquots (100 *μ*L) were mixed with 200 *μ*L of distilled water containing 50 ng of Chlor-Trimeton (IS) in 10 mL centrifuge tubes, and NaOH (2 M) was added to adjust to pH 12. The samples were then extracted twice with 5 mL of diethyl ether, vortex mixed for 3 minutes. After centrifugation at 3000 rpm (1006.2 g) for 10 minutes, the organic phase was separated and evaporated in a water bath at 40°C. Dried extracts were redissolved with 100 *μ*L of mobile phase and filtered through microfiltration membranes. Then, 10 *μ*L aliquots of the samples were injected into the UPLC-MS/MS system for analysis.

### 2.5. Method Validation

#### 2.5.1. Precision and Accuracy

The LLOQ concentration(2.5 ng/mL)and three QC sample concentrations (5, 50, and 500 ng/mL) of KET and NK were used to evaluate the assay precision and accuracy. The intraday precision was calculated for all three concentrations using six determinations per concentration (on the same day), while the interday precision was measured over 3 consecutive days. The accepted criteria for each sample were that the precision and accuracy should not exceed 10%. The precision was expressed as the relative standard deviation (RSD) and the accuracy as the relative error (RE) [11].

#### 2.5.2. Extraction Recovery and Matrix Effect

The extraction recoveries of KET and NK were determined for the three QC sample concentrations (5, 50, and 500 ng/mL). The recoveries were calculated by comparing the observed peak area ratios of biological samples to those of unprocessed standard solutions at the same concentrations [12]. The matrix effects in the approach were evaluated by comparing the peak areas of the compounds in the postextraction spiked samples with those of the standard solutions [11].

#### 2.5.3. Stability

The stabilities of KET and NK in rat plasma were evaluated at three different QC concentrations using six replicates under two conditions, including at 25°C for 12 h, and three freeze and thaw cycles. Unextracted QC samples of the two compounds at 5, 50, and 500 ng/mL concentrations were kept at 25°C for 12 h to determine the stability of the compounds in rat plasma. The samples were processed and analyzed, and then, the concentrations were compared with the nominal values of the QC samples. The stabilities of the plasma samples after three freeze and thaw cycles were determined. In each cycle, the QC samples were stored at -20°C for 24 h and thawed unassisted at room temperature. When completely thawed, the samples were refrozen with 24 h. The cycle was repeated three times, and the samples were analyzed after the third cycle. Stability was assessed by comparing the mean concentration of the stored QC samples with the mean concentration of freshly prepared QC samples [11].

### 2.6. Animal and Pharmacokinetic Study

The Sprague-Dawley rats (220–250 g) were randomly assigned to one of the three groups: the saline group, KET group, or KET combined with *l-*THP group (each, *n* = 6). KET and *l-*THP were dissolved in saline. Gastric gavage was used for administration of 20 mL/kg saline to the saline group, 100 mg/kg KET to the KET group [13], and 100 mg/kg KET and 40 mg/kg *l-*THP to the KET combined with *l-*THP group. Blood samples (0.5 mL) were withdrawn from the orbital vein at 0.083, 0.25, 0.5, 0.75, 1, 1.5, 2, 3, 4, and 5 hours after drug administration. The doses of the drugs and sampling intervals were chosen based on previous reports [13, 14]. All samples were collected into heparinized tubes and then centrifuged at 13,000 rpm (14900 g) for 10 minutes at 4°C. The plasma was transferred to fresh tubes and stored at −80°C prior to analysis.

### 2.7. Pharmacokinetics Parameters and Statistical Analysis

The plasma concentration versus time data for each rat was analyzed using DAS (Drug and Statistics) software (version 2.0, China Pharmaceutical University). The area under the curve from time zero until infinity (AUC_0-∞_), distribution phase half-life (*t*_1/2*α*_), elimination phase half-life (*t*_1/2*β*_), maximum plasma concentration (*C*_max_), absorption phase half-life (*t*_1/2a_), time of maximal plasma concentration (*t*_max_), and clearance (CL) were estimated using one- and two-compartment model calculations performed with DAS software. Comparisons between parameters were performed using *t*-test. In all analyses, *P* < 0.05 (two tailed) was taken to indicate statistical significance.

## 3. Results

### 3.1. UPLC-MS/MS Method Verification

#### 3.1.1. Selectivity

**Figure 3:**
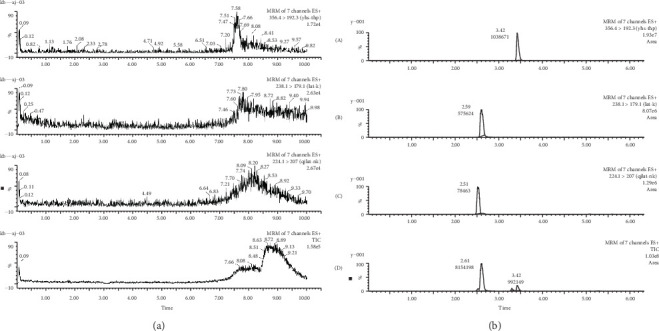
Representative ultraperformance liquid chromatography tandem mass spectrometry (UPLC-MS/MS) chromatograms: (a) blank plasma and (b) a rat plasma sample after intragastric administration of (A) *l*-tetrahydropalmatine (*l-*THP), (B) ketamine (KET), and (C) norketamine (NK).

Typical UPLC-MS/MS chromatograms of blank plasma and plasma samples collected from the orbital vein of rats are shown in Figure 3, demonstrating that there were no major interference from endogenous substances in the analysis of the compounds, and good selectivity was achieved.

#### 3.1.2. Linearity and Sensitivity

Calibration curves of KET and NK in rat plasma indicated good linearity over the concentration range 2.5–500 ng/mL. The regression equations were as follows: *Y*_1_ = 0.0009*X*_1_ + 0.0034, *r* = 0.9995 and *Y*_2_ = 0.0009*X*_2_ − 0.0015, *r* = 0.9998. *Y*_1_ represents the ratio of the peak intensity of KET to the internal standard, and *X*_1_ represents the concentration of KET in plasma; *Y*_2_ represents the ratio of the peak intensity of NK to the internal standard, and *X*_2_ represents the concentration of NK in plasma. The assay had an LLOQ of 2.5 ng/mL for KET and NK in rat plasma samples. The results are shown in Table 2.

#### 3.1.3. Precision and Accuracy

Table 3 summarizes the KET and NK analysis results. The RSDs for the intraday and interday precision at three concentration levels of KET were below 3.34% and 4.31%, respectively. The accuracy of KET was in the range of -8.11% to -1.41%. While the RSDs for the intraday and interday precisions of NK were below 5.75% and 6.53%, respectively. The accuracy of NK was in the range of -9.28% to -6.07%. These results indicated that the precision and accuracy of the established UPLC-MS/MS method were satisfactory for KET and NK.

#### 3.1.4. Extraction Recovery and Matrix Effect

The extraction recoveries of KET were no less than 81.23 ± 3.45%, while the extraction recoveries of NK were no less than 80.42 ± 4.57% (Table 3), suggesting that the precision and accuracy of this method were acceptable. The matrix effects of KET at the three QC levels ranged from 90.61 ± 3.22% to 105.47 ± 2.89%, while the matrix effects of NK ranged from 91.38 ± 2.56% to 104.35 ± 3.21%, which indicated that the matrix effect was negligible.

#### 3.1.5. Stability

The results for the room temperature and freeze-thaw stability are shown in Table 4. There was no major degradation of the two compounds under the different storage conditions.

### 3.2. Pharmacokinetic Study

**Figure 4:**
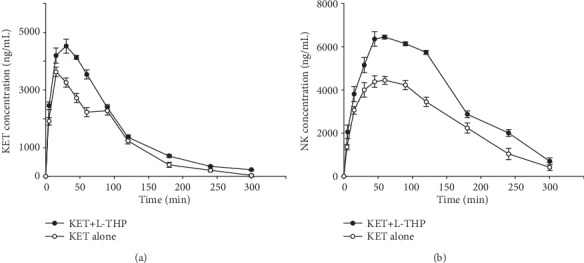
Mean (a) KET and (b) NK concentration–time curves of plasma levels after administration of KET alone and in combination with *l-*THP (*n* = 6).

The mean plasma concentration–time curves of KET and NK are presented in Figure 4. The pharmacokinetic parameters for KET and NK are listed in Tables 5 and 6. When administered intragastrically to rats, KET was absorbed rapidly; with a short *t*_max_ of 15.0 min. Concomitant intake of l-THP resulted in a significant increase in the plasma KET *C*_max_ and AUC. The mean KET *C*_max_ value increased from 3.63 ± 2.40 *μ*g/mL to 4.50 ± 1.31 *μ*g/mL (*P* < 0.05) and the mean AUC from 358.75 ± 3.21 *μ*g·min/mL to 512.75 ± 4.70 *μ*g·min/mL (*P* < 0.05). Furthermore, when l-THP was coadministered, the plasma NK *C*_max_ and AUC increased from 4.45 ± 1.71 *μ*g/mL to 6.44 ± 2.62 *μ*g/mL (*P* < 0.05), and from 803.42 ± 3.70 *μ*g·min/mL to 1176.33 ± 3.80 *μ*g·min/mL, respectively (*P* < 0.05).

## 4. Discussion

A sensitive, rapid, and selective UPLC-MS/MS method was established in the present study to determine the concentrations of KET and NK in rat plasma. The method was used successfully to study the pharmacokinetic effects of *l-*THP on KET in rat plasma.

As a DA receptor antagonist, *l-*THP has therapeutic potential in the treatment of addiction to many drugs, including cocaine [15], heroin [16], and methamphetamine [17]. In our previous study, *l-*THP showed a potential therapeutic effect on KET addiction [10]. However, the pharmacokinetic effects of *l-*THP on KET remained unclear. Chen et al. studied the pharmacokinetics interaction between KET and rhynchophylline using UPLC-MS/MS. They found that there was a reciprocal inhibition between KET and rhynchophylline [18].

When various drugs are used together, attention must be paid to the interactions between them. Chinese herbal medicine can induce or inhibit hepatic drug-metabolizing enzymes (CYP450), so there may be metabolic interactions between these agents and Western medicines [19].

Chinese medicine can inhibit or induce enzymes, such as CYP450, drug transport proteins, and UDP-glucuronosyl transferase (UGT), thereby influencing the concentrations of medications in blood, body fluids, and tissues, which would in turn alter the pharmacokinetic parameters [18–21].

The primary pharmacokinetic parameters of KET were as follows: *C*_max_, 3.63 ± 2.40 *μ*g/mL; AUC, 358.75 ± 3.21 *μ*g·min/mL; *t*_max_, 15.0 ± 1.20 min; *t*_1/2_, 31.42 ± 1.59 min; and CL, 3.36 ± 1.01 L/h/kg. Compared to the KET alone group, there were significant differences in *C*_max_, AUC, and *t*_max_ after administration of KET combined with *l-*THP. *C*_max_, AUC, and *t*_max_ were increased by 1.42-, 1.24-, and 2.0-fold, respectively, after combined use, suggesting that *l-*THP may increase the absorption of KET. The primary pharmacokinetic parameters of NK were as follows: AUC, 803.42 ± 3.70 *μ*g·min/mL and *C*_max_, 4.45 ± 1.71 *μ*g/mL. Compared to the KET alone group, there were significant differences in AUC and *C*_max_ after administration of KET combined with *l-*THP. AUC and *C*_max_ were increased by 1.46- and 1.44-fold, respectively, after combined use, suggesting that *l-*THP may promote the metabolism of KET. Peltoniemi et al. reported that KET undergoes oxidative metabolism, mainly to NK, by CYP4503A and CYP2B6 [22]. Hijazi and Boulieu reported that the subtypes of CYP450 (CYP2C9, CYP2B, and CYP3A) participated in *N*-demethylation of KET in the rat liver and that KET may have an effect on the enzyme substrate *in vivo* [23]. Therefore, *l-*THP may promote the expression of CYP2C9, CYP2B6, and CYP3A4, thereby accelerating the metabolism of KET, as indicated by our results. However, further studies are needed to verify the interaction of KET with *l-*THP.

## 5. Conclusion

A UPLC-MS/MS method was established in the present study to investigate the pharmacokinetic effects of *l-*THP on KET and NK. The results showed that *l-*THP can increase the absorption and metabolism of KET, possibly through effects on hepatic enzymes. However, further studies are needed to determine the mechanisms underlying the putative interactions.

## Figures and Tables

**Table 1:** MRM parameters for drugs and IS.

Drugs	Parent ion	The cluster voltage (V)	Daughter ion	Collision energy (eV)
KET	238.1	40	179.1^∗^	25
			125.1	40
NK	224.1	40	207.1^∗^	19
			125.1	32
IS	275		230^∗^	25

^∗^Quantitative ion.

**Table 2:** Calibration equation and limits of detection of KET and NK in rat plasma.

	Drugs	Calibration equation	LLOQ (ng/mL)	Correlation Coefficient (*r*)	Linearity range (ng/mL)
Plasma	KET	*Y*_1_ = 0.0009*X*_1_ + 0.0034	2.5	0.9995	2.5-500
	NK	*Y*_2_ = 0.0009*X*_2_ − 0.0015	2.5	0.9998	2.5-500

**Table 3:** Precision, accuracy, recovery, and matrix effect of KET and NK in rat plasma (*n* = 6).

Compound	Concentration ng/mL)	Precision (RSD %)		Accuracy (RE%)		Extraction recovery (%)	Matrix effect (%)
		Intraday	Interday	Intraday	Interday		
KET	LLOQ (2.5)	3.15	2.95	-4.31	-5.54	—	—
	LQC (5)	2.72	4.31	-1.41	-8.11	81.23 ± 3.45	90.61 ± 3.22
	MQC (50)	3.34	2.60	-6.43	-5.23	83.78 ± 4.87	91.78 ± 4.76
	HQC (500)	2.62	2.82	-5.92	-6.51	87.56 ± 6.92	105.47 ± 2.89

NK	LLOQ (2.5)	4.38	5.12	-6.27	-7.11	—	—
	LQC (5)	5.75	6.53	-8.46	-7.02	85.47 ± 2.63	91.38 ± 2.56
	MQC (50)	3.71	4.41	-7.43	-9.28	80.42 ± 4.57	93.44 ± 4.63
	HQC (500)	2.50	2.66	-6.28	-6.07	82.36 ± 3.66	104.35 ± 3.21

RSD: relative standard deviation; RE: relative error; LLOQ: lower limit of quantitation; LQC: low-quality control; MQC: medium-quality control; HQC: high-quality control.

**Table 4:** Stability of KET and NK in rat plasma at room temperature or freeze thaw.

Compound	Concentration (ng/mL)	12 h measured C (ng/mL)	RSD (%)	Freeze thaw	RSD (%)
KET	LQC (5)	4.71 ± 0.02	3.96	4.79 ± 0.23	5.28
	MQC (50)	48.21 ± 1.23	5.98	48.99 ± 1.54	2.31
	HQC (500)	490.31 ± 1.08	2.13	489.30 ± 2.41	3.56

NK	LQC (5)	4.86 ± 2.05	3.50	4.81 ± 0.34	4.54
	MQC (50)	48.52 ± 1.06	5.29	47.63 ± 1.67	3.56
	HQC (500)	495.81 ± 1.08	2.81	492.42 ± 3.78	4.98

LQC: low-quality control; MQC: medium-quality control; HQC: high-quality control.

**Table 5:** Pharmacokinetic parameters of KET after administration of KET alone and in combination with *l-*THP (*n* = 6).

Parameters	Unit	KET group	Parameters	Unit	KET+*l*-THP group
AUC_0-∞_	*μ*g·min/mL	358.75 ± 3.21	AUC_0-∞_	*μ*g·min/mL	512.75 ± 4.70^∗^
*t*_1/2_	min	31.42 ± 1.59	*t*_1/2*α*_	min	39.34 ± 1.12
CL	L/h/kg	3.36 ± 1.01	*t*_1/2*β*_	min	69.32 ± 2.0
*C*_max_	*μ*g/mL	3.63 ± 2.40	CL	L/h/kg	2.34 ± 1.62
*t*_max_	min	15.0 ± 1.20	*C*_max_	*μ*g/mL	4.50 ± 1.31^∗^
		—	*t*_1/2a_	min	11.27 ± 2.33
		—	*t*_max_	min	30.0 ± 1.50^∗^

Values represent the mean ± SEM of six rats/group. Through gastric gavage, rats were administered KET alone (100 mg/kg) or in combination with oral *l-*THP (40 mg/kg). ^∗^Significantly different from KET-treated rats (*P* < 0.05).

**Table 6:** Pharmacokinetic parameters of NK after administration of KET alone and in combination with *l-*THP (*n* = 6).

Parameters	Unit	KET group	KET+*l*-THP group
AUC	*μ*g·min/mL	803.42 ± 3.70	1176.33 ± 3.80^∗^
*t*_1/2_	Min^−1^	45.36 ± 1.59	47.52 ± 2.13
CL	L·kg/h	1.5 ± 0.61	1.02 ± 0.50
*C*_max_	*μ*g/mL	4.45 ± 1.71	6.44 ± 2.62^∗^
*k*_a_		0.017 ± 1.05	0.016 ± 1.20

Values represent the mean ± SEM of six rats/group. Rats were treated with oral KET (100 mg/kg) alone or in combination with *l-*THP p.o. (40 mg/kg). ^∗^Significantly different from KET-treated rats (*P* < 0.05).

## Data Availability

The data used to support the findings of this study are available from the corresponding author upon request.
